# Transcriptomic analysis of a 3D blood–brain barrier model exposed to disturbed fluid flow

**DOI:** 10.1186/s12987-022-00389-x

**Published:** 2022-11-24

**Authors:** Nesrine Bouhrira, Brandon J. DeOre, Kiet A. Tran, Peter A. Galie

**Affiliations:** Department of Biomedical Engineering, Rowan University, 201 Mullica Hill Rd, Glassboro, NJ USA

**Keywords:** Fluid dynamics, Blood–brain barrier, RNA sequencing

## Abstract

Cerebral aneurysms are more likely to form at bifurcations in the vasculature, where disturbed fluid is prevalent due to flow separation at sufficiently high Reynolds numbers. While previous studies have demonstrated that altered shear stress exerted by disturbed flow disrupts endothelial tight junctions, less is known about how these flow regimes alter gene expression in endothelial cells lining the blood–brain barrier. Specifically, the effect of disturbed flow on expression of genes associated with cell–cell and cell–matrix interaction, which likely mediate aneurysm formation, remains unclear. RNA sequencing of immortalized cerebral endothelial cells isolated from the lumen of a 3D blood–brain barrier model reveals distinct transcriptional changes in vessels exposed to fully developed and disturbed flow profiles applied by both steady and physiological waveforms. Differential gene expression, validated by qRT-PCR and western blotting, reveals that lumican, a small leucine-rich proteoglycan, is the most significantly downregulated gene in endothelial cells exposed to steady, disturbed flow. Knocking down lumican expression reduces barrier function in the presence of steady, fully developed flow. Moreover, adding purified lumican into the hydrogel of the 3D blood–brain barrier model recovers barrier function in the region exposed to fully developed flow. Overall, these findings emphasize the importance of flow regimes exhibiting spatial and temporal heterogeneous shear stress profiles on cell–matrix interaction in endothelial cells lining the blood–brain barrier, while also identifying lumican as a contributor to the formation and maintenance of an intact barrier.

**Supplementary Information:**

The online version contains supplementary material available at 10.1186/s12987-022-00389-x.

## Introduction

Steady and oscillatory shear stress affect the endothelial transcriptome, methylome, and proteome [1–5]. The context of previous studies interrogating the effects of shear stress exerted by disturbed flow is the pathogenesis of atherosclerosis; establishing a link between atheroprone regions of the vasculature and separated flow, which is characterized by low, oscillatory shear stress [6]. Ligation models provide a means to evaluate altered shear stress within animal models by either diverting flow to increase or decrease shear stress magnitude, or by inducing disturbed blood flow at specific points in the vasculature [7]. However, modifying blood flow in this way affects transport and potentially confounds the effects of shear stress on blood vessels. Two-dimensional in vitro models provide an alternative means of directly controlling the shear stress applied to endothelial cells. However, much of this work is conducted on tissue culture plastic, which provides a substantially artificial microenvironment compared to in vivo vasculature. Microfluidic chip-based systems have incorporated cell-ECM interaction [8], but the geometries used to interrogate endothelial gene expression are still primarily 2D in nature and neglect the impact of substrate mechanics. Recent advances in patterning 3D geometries facilitate flow separation in topologies mimicking in vivo vasculature [9], and therefore provide a means to evaluate the endothelial transcriptome under disturbed flow in a controlled and physiologically relevant microenvironment.

Similar to atherosclerotic plaques, the incidence of cerebral aneurysms is higher in areas of disturbed shear stress, mostly adjacent to bifurcations in regions of sufficiently high Reynolds number [10]. However, endothelial cells lining cerebral vasculature feature an increased expression of tight junctions that contribute to the blood–brain barrier, and are thus phenotypically distinct from the endothelial cells lining atherosclerotic plaques. The blood–brain barrier is present in multiple levels of vascular architecture, from pial arteries and penetrating arteries to arterioles and capillaries in the parenchyma of the brain [11]. In the microvasculature, the endothelial tight junctions are one component of the neurovascular unit, which consists of surrounding pericytes, astrocytes, microglia, and neurons and features a robust basement membrane. A recent study demonstrated that the presence of these components alters the endothelial transcriptome [12], but little is known about how fluid dynamics influence barrier endothelial cells. Transcriptional analysis indicates heterogeneous endothelial phenotypes along the different architecture of brain vasculature [13], and fluid mechanics studies have indicated that the shear stress experienced by endothelial cells in the brain varies widely [14]. Furthermore, an in vitro study suggests that shear heterogeneity in vascular branches affects endothelial function [15]. Taken together, these previous findings suggest that complex fluid flow regimes alter gene expression by endothelial cells that comprise the blood–brain barrier.

Previous computational studies have suggested that aneurysm formation is mechanically mediated, based on the altered shear stress present in the locations where saccular aneurysms are likely to form [16, 17]. Specifically, in vivo studies involving the creation of new branch points have demonstrated that high gradients of shear stress are associated with the destructive ECM remodeling that underlies aneurysm initiation [18]. Multiple studies have demonstrated that shear stress mechanotransduction not only affects cell–cell junctions, but also the feedback between cells and the surrounding extracellular matrix. Shear stress applied to cerebral endothelial cells can disrupt the balance between matrix metalloproteinases (MMPs) and tissue inhibitors of metalloproteinases (TIMPs) [19], and flow mechanotransduction also alters the structure and integrity of the blood–brain barrier through secretion of matrix protein Fgfbp1 [20]. Both of these studies provide specific examples of how fluid flow can alter cell–matrix interaction, but transcriptomics can facilitate a broader understanding of how shear stress alters endothelial interactions with the surrounding extracellular matrix. Therefore, the purpose of this study is to probe the effect of disturbed and fully developed flow applied by steady and physiological waveforms on endothelial transcription to provide insight into how complex fluid flow dictates the endothelial response.

## Methods

### Microfabrication of microfluidic device

Polydimethylsiloxane (PDMS) microfluidic devices were manufactured by soft lithography using a previously described protocol [21]. Briefly, SU-8 2025 photoresist (MicroChem, catalog 2025-2100) was deposited on a silicon wafer and exposed to a UV source through a mask with the desired geometry. The flow inlet was positioned at an angle of 45 degrees to induce separated flow within the range of Reynolds numbers appropriate for modeling arterial blood flow (Re = 50-200). The hydrogel reservoir had dimensions of 6.5 × 6.5 mm and a height of approximately 2 mm. Sylgard 184 PDMS (Ellsworth Adhesives, catalog 184) was mixed at the recommended 10:1 ratio with curing agent and used to cast negative and positive molds of the design and to pattern devices on 22 mm  ×  40 mm glass cover slips. Devices were then cured at 60 C overnight. Prior to adding a hydrogel to the device, the reservoir was filled with 5 M sulfuric acid (Sigma, catalog 339741), washed with distilled water, and incubated in dilute type I collagen (MP Biomedical, catalog MFCD00130825) (20 μg/mL) to facilitate hydrogel attachment to the PDMS [22]. The devices were then sterilized for 30 min using short wavelength UV prior to channel seeding.

### Cell culture and vessel fabrication

Prior to seeding into the microfluidic device, three different cell types were thawed and expanded. Normal human astrocytes (NHA) (Lonza, catalog CC-2565) were thawed at passage 5 and cultured for 10 days in astrocyte growth medium (AGM) (Lonza, catalog CC-3186). Human Coronary Arterial Smooth Muscle Cells (HCASMC) (Sigma, catalog 350-05A) were also thawed at passage 5 and cultured in smooth muscle growth medium (Sigma, catalog 311-500) until confluency, then passaged and cultured in 6 well plates using smooth muscle cell differentiation medium (Sigma, catalog 311D-500) for 10 days prior to vessel fabrication. Additionally, human cerebral microvascular endothelial cells (HCMEC/D3) received at passage 19 (gifted from Dr. Robert Nagele’s laboratory) were cultured and expanded on tissue culture plates coated with 1% gelatin (Sigma, catalog 1288485) in EBM-2 (Lonza, catalog CC-3156) modified with CD lipid concentrate (Life Technologies, catalog 11905031), HEPES buffer (Sigma, catalog 83264), 10% fetal bovine serum (VWR, catalog 97068-085), 5 μg/mL ascorbic acid (Sigma, catalog A92902), 1 ng/mL of bFGF (Peprotech, catalog 100-18B), 1 μM hydrocortisone (Sigma, catalog H0888), and 1% penicillin/streptomycin (VWR, catalog 97062-806), consistent with a previously described protocol [23]. The vessel seeding protocol consists of three different steps. First, NHA were seeded at a density of 1 million cells per mL in a 10 mg/mL collagen, 1.33 mg/mL hyaluronan (HA) (Sigma, catalog H388) and 1 mg/mL Matrigel (Corning, catalog 354234) hydrogel formulation used in a previous study [24]. After injecting 100 μL of the astrocyte-seeded hydrogel directly into the reservoir, an 18-g needle coated with 0.1% Bovine Serum Album (BSA) (Sigma, catalog 05470) was inserted into the device through a needle guide. After waiting 20 min to allow for gel polymerization, AGM was added on the top of the device to maintain cell viability. After an additional 20 min, the needle was pulled leaving a cylindrical void. HCASMC were then seeded into a 10 mg/mL collagen at a density of 1 million cells per mL. In lumican add-in studies, these hydrogels were supplemented with 10 μg/mL lumican (VWR, catalog PRSI96-526). A 20-g needle coated in 0.1% BSA was inserted inside the void left by the 18-g needle. After polymerization, the 20-g needle was removed leaving a final cylindrical void and surrounding annulus with a 944 μm diameter. In the final step, HCMEC/D3 (passages 22–24) were resuspended in EGM-2 at a density of 5 million per mL, then 15 μL of the HCMEC/D3-containing media was injected into the cylindrical void and incubated for 10 min, yielding a seeding density of approximately 150,000 cells per cm^2^. This process was repeated for an additional 10 min after inverting the device to assure uniform lumen coverage throughout the vessel. Devices were then submerged in EGM-2 media and left in static conditions for 2 days to ensure cell spreading and viability prior to exposure to flow or static conditions. For the flow experiments, silicone grease (Sigma, catalog Z273554) was used to close the needle guides to prevent flow leakage.

### Perfusion system setup

The peristaltic pump system was controlled using a previously described Arduino-based system [9]. A peristaltic motor (Welco, catalog WPX1-P3/32M2-CP) was controlled by an Arduino Uno (Arduino, catalog A000066) and a motor board (Ada Fruit, catalog 1438). The flow rate magnitude was controlled by using the Arduino to regulate DC voltage powering the motor. A Sensiron flow sensor was used in-line of the perfusion system to monitor the steady flow rate and pulsations. To mimic the arterial pulsation, the pulsatile flow system was designed by superposition of oscillations with the constant mean flow driven by the peristaltic pump using a previously described method [25]. The code to control the pump was written and compiled in Arduino IDE using open source libraries available within the IDE (the code is available upon request). To minimize pulsations in the system, a dampener was placed in series with the vessel. The system included a damper placed in series with the channel, to eliminate the oscillations coming from the peristaltic pump, and a Linmot^®^ linear motor (G&G Technical, catalog PS01-23 × 160H-HP-R), which was programmed to generate the flow waveform via displacing a 5 mL syringe connected to the actuator system using the LinMot software (open source) [26]. Corsair 120 mm 12v fans (Best Buy, SKU 5845209) were mounted to the linear motor to regulate the temperature and prevent the system from overheating.

Two vessels were run in parallel for each perfusion experiment. For steady flow, the linear motor was removed from the flow circuit and the peristaltic pump was set to a flow rate of 8 mL/min to be consistent with a previous study that validated the presence of disturbed flow using a combination of computational fluid dynamics and microparticle image velocimetry [9]. In the steady condition, the shear stress range in the disturbed region varied by approximately 500 Pa/mm. The fully developed shear stress was approximately 15 dyn/cm^2^ in the steady condition. The pulsatile flow rate varied between 1 and 8 mL/min, consistent with a previous study on implementing a physiological waveform within this 3D blood–brain barrier bifurcation model [25]. Due to the pulsatile nature of the flow, the shear stress within the disturbed region varied substantially. The maximum shear stress in the fully developed region was approximately 15 dyn/cm^2^. These values are consistent with a measured middle cerebral artery shear stress of 20 dyn/cm [2, 27]. Following perfusion, vessels were either placed in fixative for immunocytochemistry or perfused with FITC-dextran to measure permeability.

### Immunocytochemistry

Vessels exposed to either flow or static conditions were fixed in 4% paraformaldehyde (Alfa Aesar, catalog J61899) for 30 min at room temperature at the end of the experiment. Following fixation, the top layer of the microfluidic device was separated using a razor blade, and the hydrogel containing the vessel was then gently removed from the device. Immunofluorescence was performed after blocking the gel in 3% BSA for 30 min at room temperature and incubating overnight at 4 C in a primary antibody for either zonula occludin-1 (ZO1) (1:250, Cell Signaling Technology, 8193), glial fibrillary acidic protein (GFAP) (1:100, Santa Cruz, sc-166481), alpha smooth muscle cell actin (1:100, ThermoFisher, 41-9760-80), or lumican (1:100, Santa Cruz, sc-166871). Gels were then washed three times with PBS for 5 min and incubated in the appropriate secondary antibody (1:500, Santa Cruz, assorted), 1:500 Hoechst (Life Technologies, catalog 62249), and FITC-phalloidin (Life Tech, F432) for 1 h at 37 C. Confocal stacks were acquired using a Nikon A-1 confocal scanning microscope.

### Permeability testing

At the completion of the 24-h experiment, the microfluidic devices were placed on the stage of an inverted epifluorescent (Nikon Ti-E) and perfused with 4 kDa FITC-dextran (Sigma, catalog 46944) at a flow rate of 10 μL/min using a linear syringe pump (Kent Scientific, catalog 13005104) over the span of 10 min. This flow rate was chosen to avoid any flow separation and assure fully developed flow throughout the channel. Images were taken at 30-s intervals and then imported into ImageJ for analysis. The permeability coefficient was determined using the following Equation [28]:

1 $$P = \frac{di}{{dt}}\frac{r}{{2l_{0} }}$$

where $\frac{di}{{dt}}{ }$ is the change in florescent intensity of the region of interest outside the lumen, r is the lumen radius (472 μm) and I_0_ is the maximum intensity in the lumen during the test. Permeability was measured at two separate locations corresponding to fully developed flow and the near side wall disturbed flow (separated flow).

### RNA sequencing

To measure gene expression profiles in the different flow regimes, channels were removed from the microfluidic devices after 24 h of steady or pulsatile flow perfusion and sectioned into disturbed, impinged, and fully developed regions based on their geometry using a razor. For each region, the endothelial cell layer was separated from the rest of the gel prior to RNA extraction. Two channels for each condition were required to produce a sufficient quantity and quality (RIN > 6.0) of mRNA. The mRNA was extracted using the PicoPure RNA isolation kit (Fisher Scientific, catalog KIT0204) following manufacturer’s protocol. Twenty-one total channels were required for these experiments (three channels in duplicate for both steady and pulsatile flow besides three channels for static condition as a control). For quantitative analysis, the Nanodrop spectrophotometer was used to measure the RNA concentration and quality. Quality was indicated by measuring the absorbance ratios (260/280). Ratios around 1.8 were classified as pure and therefore acceptable for gene sequencing. The mRNA was shipped to an external company (GeneWiz) for next generation Sanger sequencing. A group of open source bioinformatics software programs (FastQC (version 0.11.9), Trimmomatic (version 0–35), Htseq (version 0.11.1), STAR (version 2.7.10b)) along with a high-performance computing cluster (16 cores, 60 GB) was used to analyze the data. FASTQC (version 0.11.9) was used to read the raw sequences and perform a quality check. All reads not complying with the quality protocol were further analyzed through Trimmomatic on the cluster to perform the trimming steps using phred + 33 quality score and paired-end data. Using the NCBI Datasets Genome page, the Fasta and gff3 files were downloaded and STAR, a well-known Genome aligner was used to align our sequence with the human reference genome sequence GRCh.38. Htseq was then used to count the aligned reads (output from STAR) per gene. Outputs from Htseq were imported as txt files into R software for statistical analysis. Quality control, preprocessing, and differential gene expression were peformed using the limma-voom analysis workflow. The disturbed, impinged, and static conditions were compared to the fully developed region as a reference.

### qRT-PCR

After perfusion, hydrogels were removed from the microfluidic device and cut into regions representative of disturbed, impingement, and fully developed regions. mRNA from the endothelial cells was isolated using PicoPure RNA Isolation kits and reverse transcribed to cDNA using qScript (Quantabio, catalog 95048). Quantitative PCR was then performed with SYBR Green reagents (Life Technologies, catalog S7563), with the primers given in Table 1 used to amplify targets. Tests were conducted in triplicate for each region.

**Table 1:** Primer sequences for endothelial qRT-PCR

Primer	Forward sequence	Reverse sequence
NONO	GTGTAGCGTCGCCGTTACTC	CCTTCATTTTGGCTGCTGGC
Lumican	TCATCCCTGGTTGAGCTGGAT	AGGATAATGGCCCCAGGATCT
MMP1	AAAATTACACGCCAGATTTGCC	GGTGTGACATTACTCCAGAGTTG
MMP3	AGGACAAAGCAGGATCACAGTTG	CCTGGTACCCACGGAACCT
MMP10	TTACATTGCTAGGCGAGATAGG	CAGTCACAGAACATGCAGGAA
MMP12	GATGCTGTCACTACCGTGGGAA	CAATGCCAGATGGCAAGGTTGG

Relative quantities (RQ) represented double normalization to Non-POU domain-containing octamer-binding protein (NONO) from a static sample. Relative expression was quantified as 2^−ΔΔC^_T_ where C_T_ is cycles to threshold.

### Lumican knockdown

Cells were plated in a 6-well plate at 200 k per well until they reached 60–80% confluency. A mixture of transfection reagent (Santa Cruz, sc-29528) and transfection media (Santa Cruz, sc-36868) was added to the transfection solution, and then to the cells following manufacturer’s protocol. Lumican siRNA (Santa Cruz, sc-44805) was added to the transfection solution and exposed to the cells for 6 h at 37C. Then, the transfection solution was removed and replaced by EGM-2 for 24 h prior to seeding. Transfection efficiency was verified by western blotting: cells were lysed in sample buffer containing DTT (Sigma, catalog 10197777001) and LDS (Sigma, catalog L9781), boiled, and loaded onto 4–12% tris–glycine gradient gels and separated with 100 V for 45–90 min. Proteins were transferred to a 0.45 µm PVDF membrane (Life Technologies, catalog 88518). Membranes were blocked and incubated with anti-lumican (1:100, Santa Cruz, sc-166871), and then visualized with HRP-conjugated secondary antibodies (1:4000, CST L27L9). Blots were then stripped by covering with a mild glycine solution (15-g glycine, 1-g SDS, and 10 mL Tween 20 in 1 L of distilled water) for 20 min (replacing the solution at 10 min), and then washing in PBS and TBST. The stripped blots were reblocked and incubated with anti-beta-actin (1:400, Santa Cruz, sc-47778) and visualized using HRP. Blots were quantified by measuring band intensity using ImageJ and normalizing relative expression to scrambled conditions.

### Monolayer experiments

For the monolayer experiments, 400 μL of the hydrogel formulation used in the annulus was sandwiched between PDMS-coated plates and a sterile 40 mm glass coverslip to create a uniform circular hydrogel. Following polymerization, the cover slip was removed and HCMEC/D3 were seeded on the polymerized gel at a density of 4 k/cm^2^ and allowed to adhere for 30 min prior to addition of EGM-2. Monolayers were incubated for 2 days in static culture to ensure confluency, then exposed to fluid shear stress. 1.5 Pa of constant fluid shear stress was applied using a 40 mm 1° cone plate on a rheometer (Waters, DHR-3) for 24-h on a Peltier plate set to 37 C to approximate the shear stress applied by steady fully developed flow. A shear stress gradient was applied using a 40 mm flat plate to mimic the shear stress profiles experienced by cells exposed to disturbed flow by using a gap of 300 μm and applying a rotation rate of 150 rad/s (approximately 500 Pa/mm). The media was supplemented with HEPES buffer to a final concentration of 10 mM to maintain pH and sterile Millipore was added to the plate during exposure of flow to counteract evaporative loss at a rate of 15 μL/min. At the end of the experiment, the gel was removed from the setup and lumican expression was measured using the same Western blotting method described above after lysing the cells in 1X lysis buffer (CST, catalog 9803).

### Turbidity tests

Turbidity tests were conducted to assess the microstructure of collagen fibril formation and organization following lumican addition. Collagen fibril formation was measured as previously described [29, 30] at 340 nm in a UV sensitive 96-well plate in a plate reader. Each assay consisted of 100 μl of collagen at 20 mg/ml and variable concentrations of recombinant human lumican using distilled water as diluent. Turbidity was measured every 2 min for 60 min. Readings were blanked against the diluent buffer and data were compared to collagen without recombinant lumican as a control. Each experiment was conducted in triplicate. Experimental turbidity curves were analyzed in terms of the maximum rate of turbidity change observed and compared to collagen without lumican.

### Rheology

To determine the impact of lumican on the mechanical properties of the ECM, Recombinant lumican was prepared and added to the collagen gel at different concentrations based on a previous study [29]. Lumican-collagen composite gels were polymerized on a strain-controlled rheometer and the viscoelastic mechanical properties of the gels were assessed as a function of lumican concentration. Both storage and loss moduli and loss angle were measured for a range of strain magnitude of 0.1% and frequency of 50 Hz, respectively. Data were compared to collagen without recombinant lumican as a control. Each experiment was conducted in triplicate. These hydrogels were also lyophilized and sputter coated for visualization with a scanning electron microscope.

### Statistical analysis

The open source statistics package, R, was used to perform all statistical analysis. Edge R package was used to determine the differentially expressed genes. PCA (Principal component analysis) was performed on the data to reduce the number of columns using the Variance, covariance, covariance matrix, eigenvalues and eigenvectors. Two sample t-tests were used to determine significance in gene expression quantification as determined by qRT-PCR, and permeability assays, with the number of vessels greater than or equal to 3.

## Results

### Effects of complex fluid dynamics on the transcriptome of blood–brain barrier endothelial cells

**Fig. 1:**
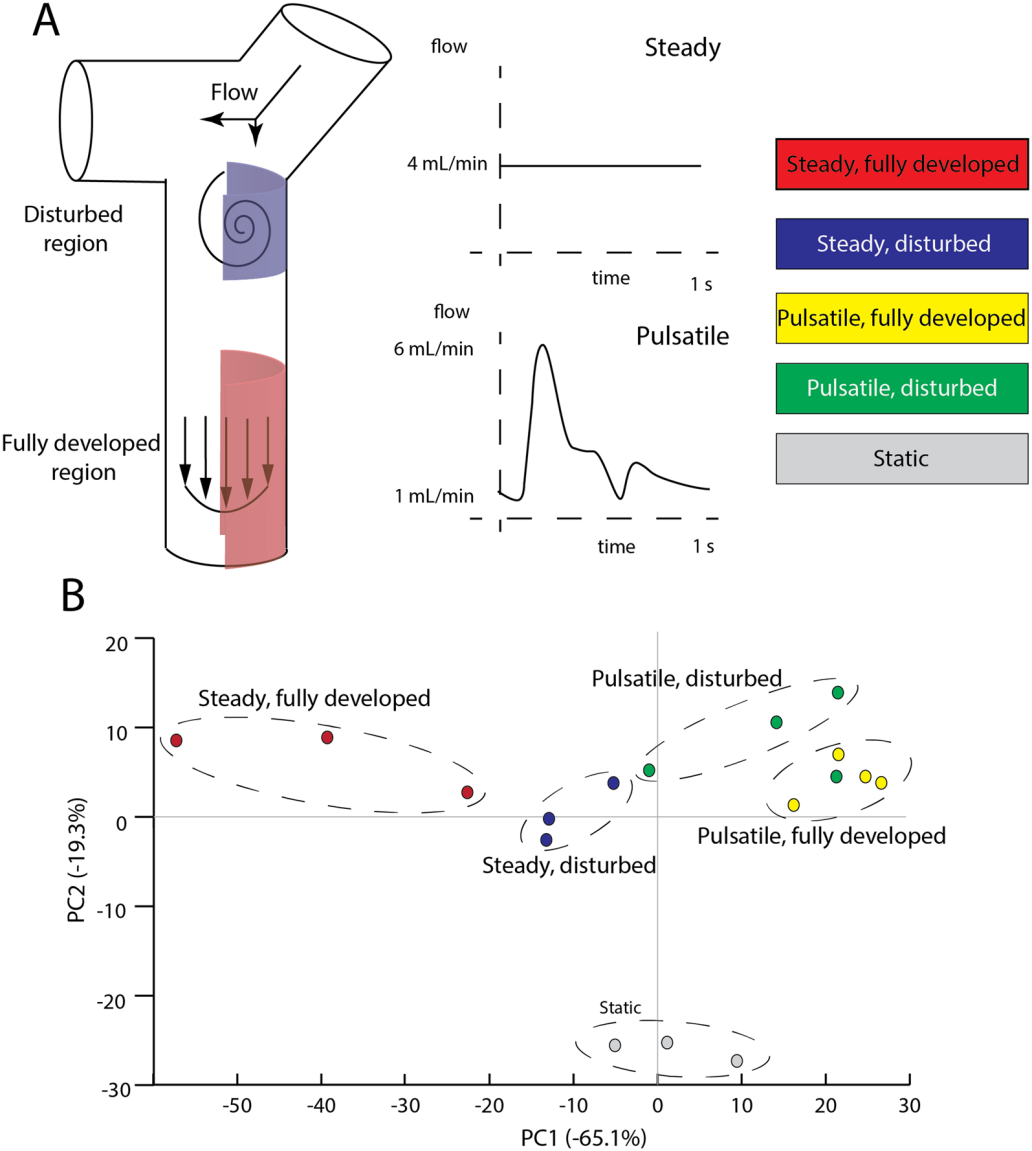
**A** Schematic of the bifurcation topology labeling the two regions where endothelial cells were isolated for sequencing as well as the two waveforms (steady and pulsatile) of flow perfused through the vessel. **B** Principal component analysis indicating clustering of flow regimes

The primary advantage of an in vitro system over animal-based models is the ability to precisely control the fluid dynamics within the vessels. The in vitro bifurcation model used here involves a co-culture of astrocytes, smooth muscle cells, and barrier-forming endothelial cells seeded within a three-dimensional architecture within a microfluidic device that facilitates a complex fluid dynamics environment. This model has been used previously to interrogate the response of the blood–brain barrier to disturbed fluid flow, characterized by separated flow, recirculation, and a shear stress gradient in the axial direction [9]. Smooth muscle cells are used because Reynolds numbers sufficient to exhibit disturbed flow only occurs in larger vessels that contain smooth muscle. The model also provides the ability to isolate cells from a single vessel in regions exposed to disturbed flow separately from those characterized by fully developed flow downstream of the bifurcation (Fig. 1A). Moreover, a previously described fluidic delivery system allows for the perfusion of either pulsatile, physiologically relevant flow waveforms or steady flow to the bifurcation model (Fig. 1A), so that the effect of time-dependent shear profiles can be evaluated. After 24 h of flow, endothelial cells were delaminated from the inside of the vessel in either the disturbed or fully developed region, and RNA sequencing measured the transcriptome of the cells. In total, five conditions: (i) steady and fully developed, (ii) steady and disturbed, (iii) pulsatile and fully developed, (iv) pulsatile and disturbed, and (v) static culture were tested.

**Fig. 2:**
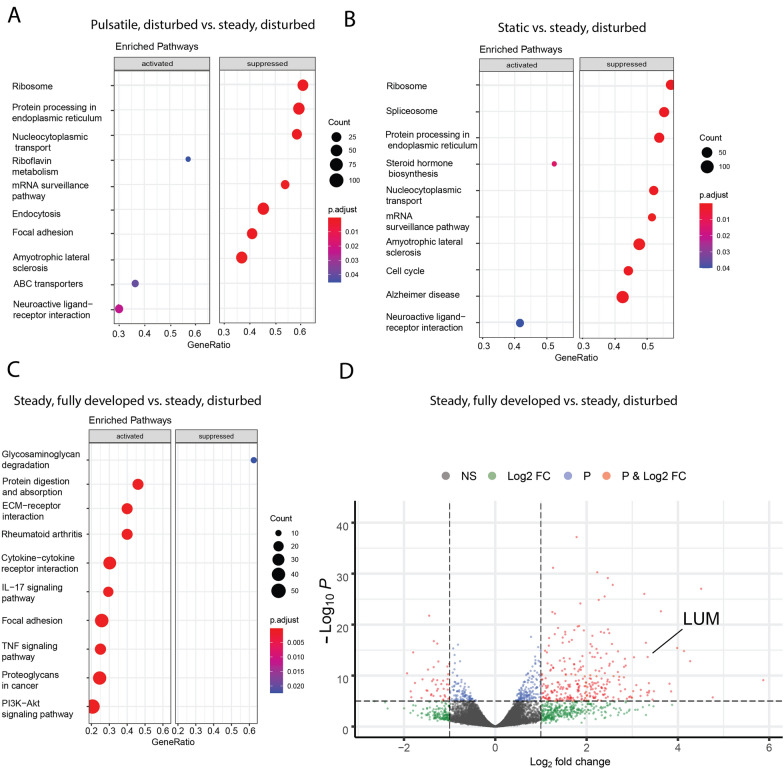
Functional enrichment analysis compared to the steady, disturbed condition for **A** pulsatile, disturbed, **B** static, and **C **steady, fully developed. **D** Volcano plot with the location of lumican indicated by LUM

Principal component analysis indicated distinct clustering of the five groups, and revealed several trends in the expression patterns of the cells exposed to different flow regimes (Fig. 1B). The flow conditions clustered from the static condition along principal component 2 (PC2), with substantial separation between the groups exposed to fluid flow regardless of the mode (fully developed/disturbed) and steadiness. The conditions resulting in barrier instability (disturbed flow for both steady and pulsatile conditions and static culture) clustered towards the center of principal component 1 (PC1). Yet, the steady and pulsatile fully developed conditions also exhibited separation along PC1, indicating that the dynamics of the flow waveform has a substantial effect on transcription patterns. Functional enrichment patterns were compared between the steady and disturbed conditions, given that these conditions exhibited the highest permeability in previous experiments using this 3D bifurcation model [9]. The pulsatile disturbed condition, which also exhibits barrier disruption though not as severely as steady disturbed [25], features increased expression of ABC transporters, which is an indicator of barrier function [31, 32] (Fig. 2A). Previous studies have also indicated that cells in regions of disturbed flow exhibit increased mitosis, and comparing the static condition to steady disturbed reveals a comparative suppression of genes associated with the cell cycle in Fig. 2B. Additionally, the steady fully developed condition had a large suppression of genes associated with glycosaminoglycan degradation compared to steady disturbed (Fig. 2C). Analysis of differentially expressed genes identified lumican, a small leucine-rich proteoglycan, as having significantly decreased expression in the steady, disturbed condition compared to steady, fully developed flow, as shown in a volcano plot in Fig. 2D. Lumican expression in human brain tissue has been previously described [33]. Table 2 summarizes the top 8 most downregulated genes in the disturbed region compared to the fully developed region in steady flow. In addition to lumican, the table includes four genes for MMPs

**Table 2:** Differential gene expression between fully developed and disturbed steady flow

Most downregulated genes in disturbed region		
Gene name	Log FC	P value
Lumican	3.247	1.187e− 50
MMP3	3.971	1.558e− 44
MMP1	2.525	6.499e− 32
MMP10	3.587	2.327e− 20
FBXO32	1.778	4.238e− 29
DNER	2.092	1.598e− 28
SERPINB2	2.363	2.690e− 28
MMP12	6.364	3.606e− 28

Taken together, these results suggested decreased lumican expression to be a direct result of disturbed flow, warranting further investigation into its effect on blood–brain barrier stability.

### Validation of lumican expression in cerebral microvascular endothelial cells

**Fig. 3:**
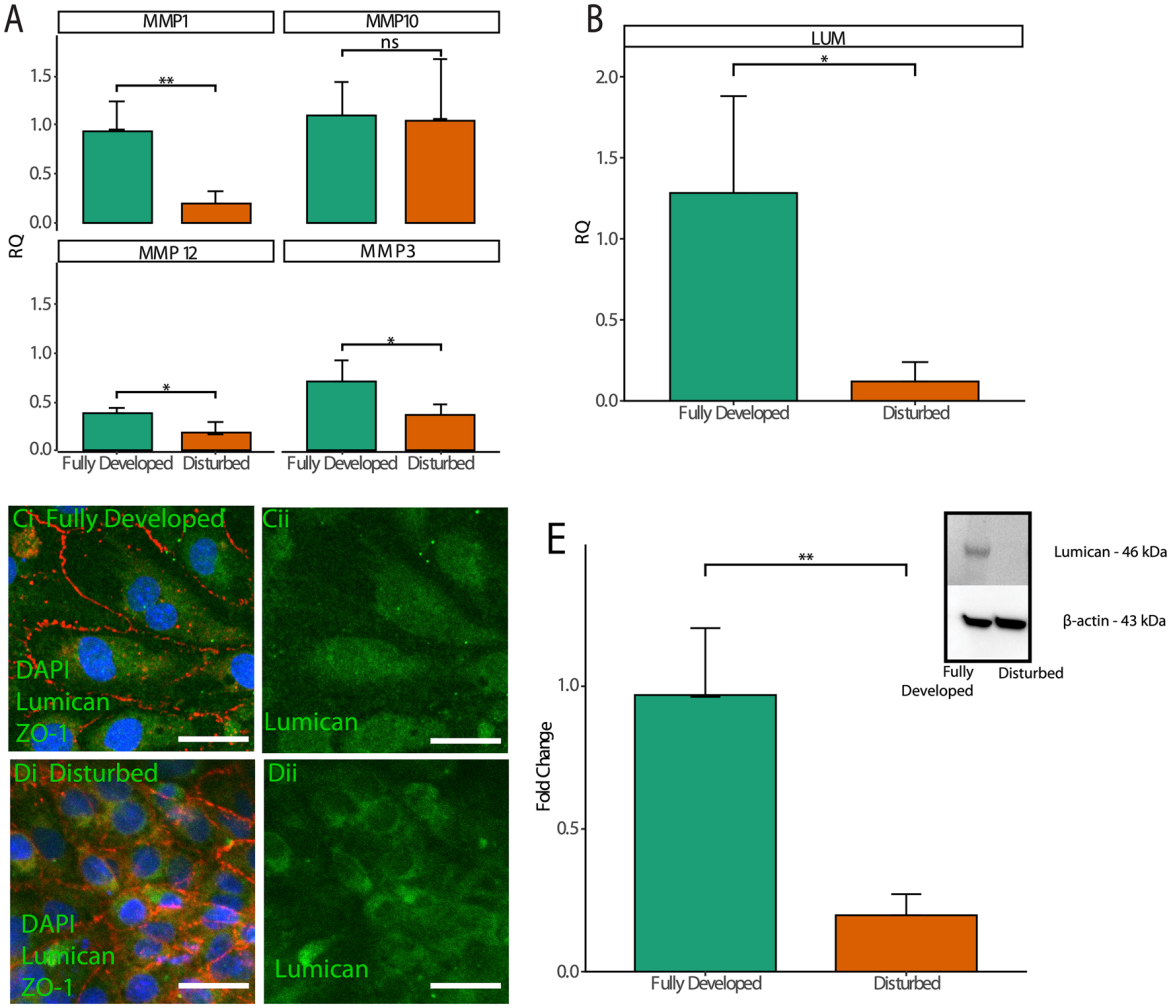
**A** qRT-PCR quantification of differentially expressed genes identified by RNA sequencing. **B** Lumican quantification between portions of the vessel exposed to steady and fully developed. **C-D** Portions of fully developed (**C**) and disturbed (**D**) regions from the 3D vessels stained with anti-ZO-1 (red) and anti-lumican (green) and DAPI (blue). Scale bar = 25 μm. **E **Protein quantification of lumican expression from monolayers exposed to uniform shear stress (“fully developed”) or a shear gradient (“disturbed”). * p < 0.05

qRT-PCR validated the results of RNA sequencing: qRT-PCR was conducted using probes for five of the eight most downregulated genes in the disturbed region including MMP1, MMP3, MMP10, MMP12, and lumican. Figure 3A indicates that all genes with the exception of MMP10 were significantly increased in the fully developed region compared to cells exposed to disturbed flow. Lumican in particular had a greater than tenfold difference in expression between fully developed and disturbed regions (Fig. 3B). Immunofluorescence was conducted in regions of both fully developed and disturbed flow within the vessel model to assess differences in lumican localization and expression. Figure 3C-D shows that lumican expression is spread diffusely throughout the cell in endothelial cells exposed to fully developed flow, whereas the expression appears less homogenous in cells from the region of the vessel exposed to disturbed flow. Western blotting was conducted to determine whether disturbed flow also caused decreased protein expression of lumican. In order to generate sufficient protein for the assay, cells were plated on a two-dimensional hydrogel and fluid shear stress was applied by a cone and plate rheometer. Additional file 1: Figure S1 indicates how a flat plate geometry on a rheometer applied a radial shear stress gradient that matched the shear gradient caused by disturbed flow within the in vitro vessel model. Figure 3E shows that the shear gradient representative of disturbed flow resulted in decreased protein expression of lumican (the full blot is shown in Additional file 1: Figure S2 to show the specificity of the antibody). This assay also suggests that the shear gradient is the characteristic of separated flow that is responsible for barrier disruption in cells exposed to disturbed flow. Overall, these assays validate that disturbed flow reduces lumican expression by cerebral microvascular endothelial cells.

### Lumican expression is needed to maintain blood–brain barrier integrity in cells exposed to fully developed flow

**Fig. 4:**
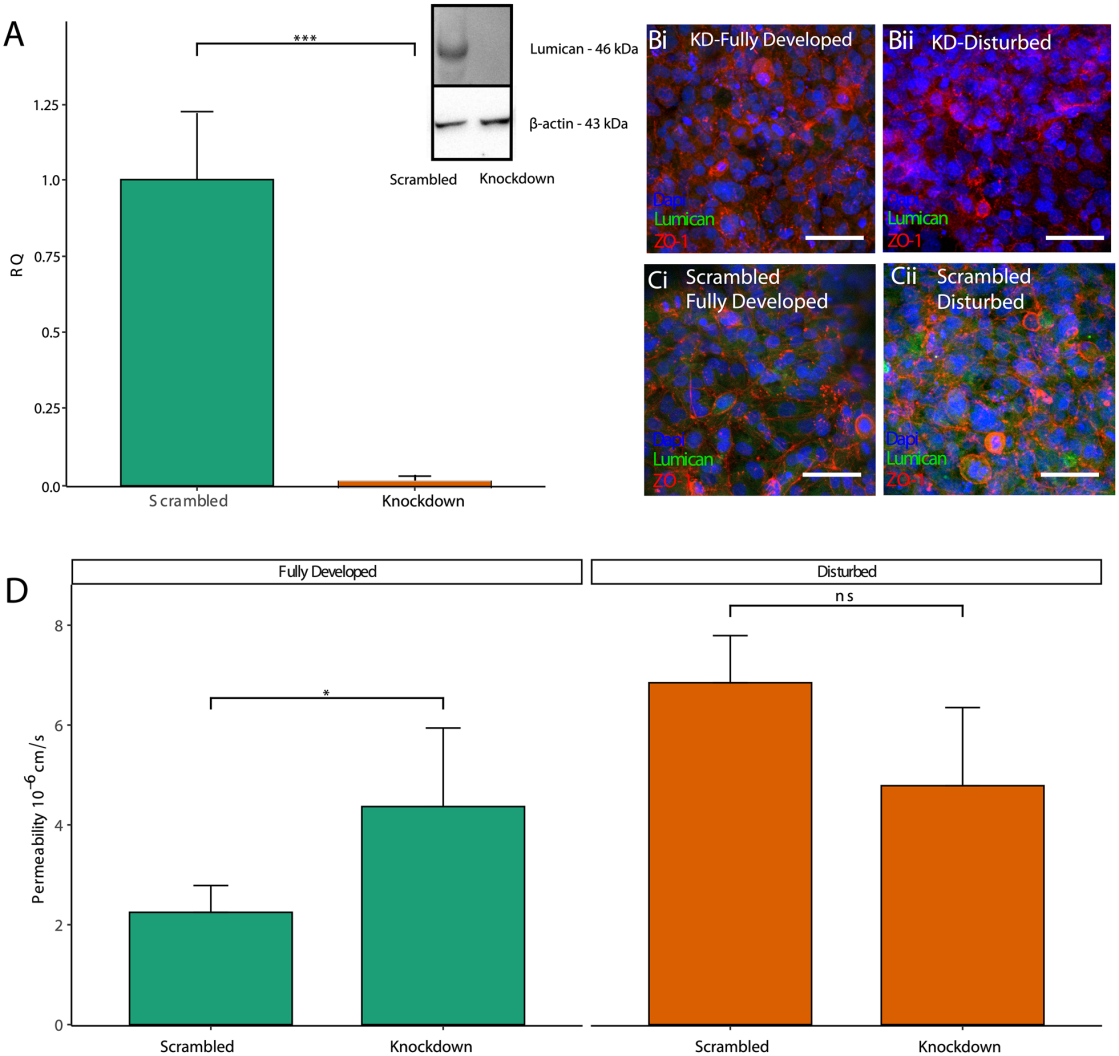
**A** Validation of lumican knockdown efficiency. **B-C** Immunofluorescence indicating the effect of lumican knockdown (**B**) on localization of ZO-1 to cell–cell junctions in both fully developed (i) and disturbed (ii) regions, with scrambled siRNA, labeled as "KD" serving as control (**C**). **D** Permeability quantification of vessels after 24 h perfusion. Scale bar = 50 μm, * p < 0.05. n = 3 per condition

Having demonstrated that a reduction in lumican expression correlated to barrier disruption in cells exposed to disturbed flow, experiments were conducted to determine whether lumican expression is necessary for maintaining barrier integrity. siRNA was used to knockdown the expression of lumican in the HCMEC/D3 cells. Figure 4A indicates that the siRNA resulted in an approximately 50% reduction in lumican expression compared to the scrambled control at three days post-transfection, which is the length of time between seeding and evaluation of barrier integrity. Bifurcation vessels were exposed to flow and immunofluorescence was used to evaluate localization of ZO-1 to the cell–cell junctions in cells treated with scrambled controls and siRNA-mediated knockdown after application of steady flow. Figure 4B shows that lumican knockdown cells exhibited reduced localization of ZO-1 to the cell–cell junctions in both fully developed and disturbed regions, indicative of barrier disruption. In contrast, cells treated with the scrambled siRNA displayed distinct differences in ZO-1 localization between fully developed and disturbed regions (Fig. 4C). In order to quantitatively measure barrier integrity, dextran permeability experiments were conducted for the scrambled and knockdown condition in both fully developed and disturbed regions of the vessel. The permeability in cells with reduced lumican expression exhibited significantly higher permeability (4.4 ± 1.6E− 6 cm/s) compared to scrambled controls (2.2 ± 0.5E− 6 cm/s) in the fully developed region (Fig. 4D). In contrast, lumican knockdown exhibited no significant difference in barrier permeability in the disturbed region (scrambled: 6.8 ± 0.9E− 6 cm/s, knockdown: 4.8 ± 1.6E− 6 cm/s) of the vessel, where the barrier integrity was already compromised by the separated flow waveforms. Given previous studies indicating that lumican can interact with intracellular small GTPase signaling, a RhoA activity assay was conducted in both the scrambled control and lumican knockdown conditions. Additional file 1: Figure S3 indicates no significant difference between the conditions, suggesting that the mechanistic effects of lumican expression do not alter RhoA activity.

### Incorporation of exogenous lumican in the extracellular matrix recovers the barrier in fully developed regions

**Fig. 5:**
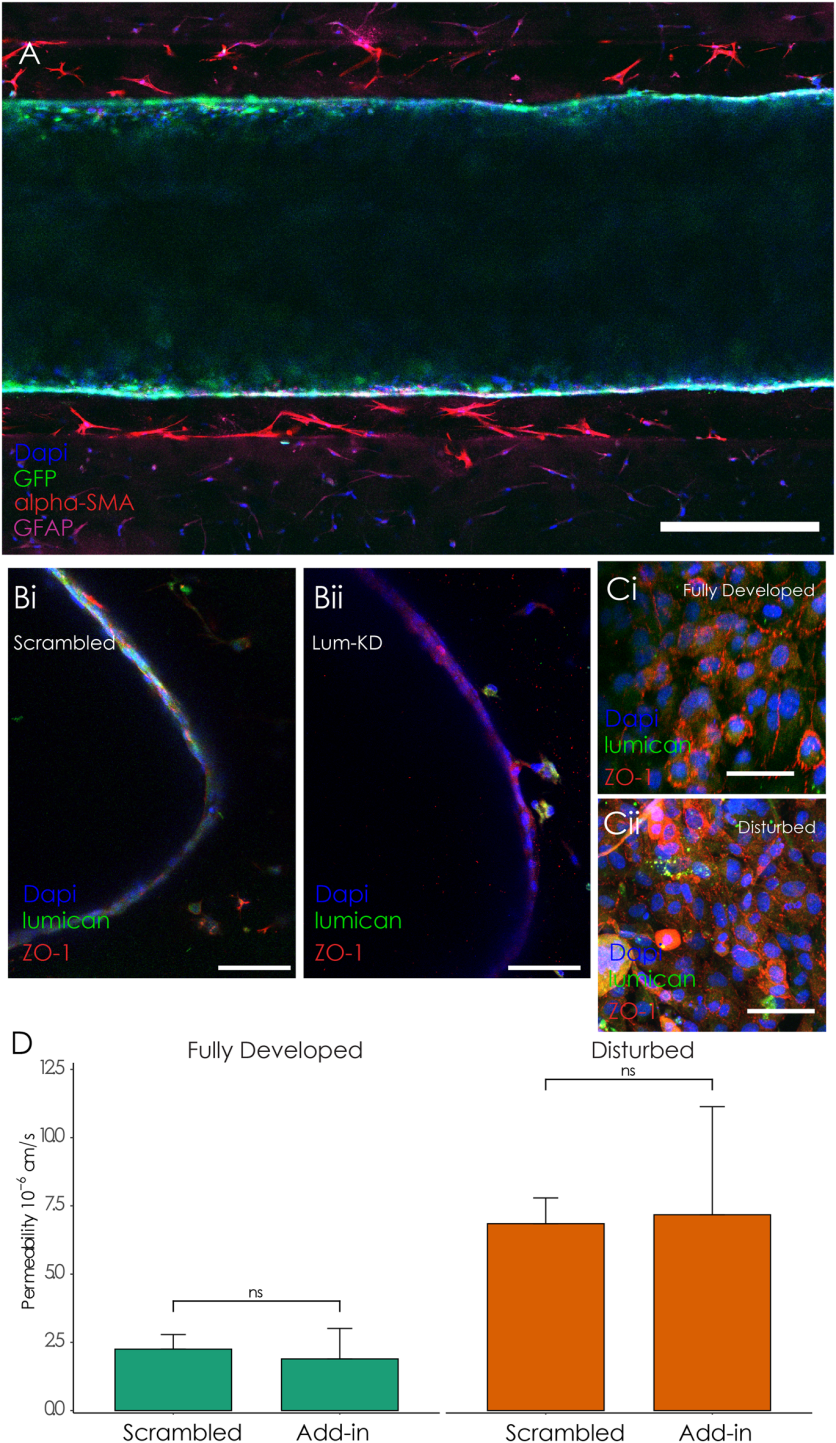
**A** Axial cross-section of the vessel showing the location of GFP-labeled endothelial cells, and smooth muscle and astrocytes stained with anti-alpha-SMA and anti-GFAP, respectively. Scale = 100 μm. **B** Radial cross-sectional images of the vessel lumens indicating differences in lumican expression between scrambled (i) and knockdown (ii) conditions. Scale bar = 100 μm. **C** ZO-1 localization to cell–cell junctions of knockdown cells with lumican added exogenously into the matrix in the fully developed (**C**,**i**) compared to the disturbed (**C**,**ii**) region. Scale bar = 25 μm. **D** Permeability quantification compared to static controls in fully developed and disturbed regions, * p < 0.05. n = 3 per condition

Given that the lumican knockdown condition exhibited barrier disruption, experiments were conducted to interrogate whether exogenous addition of lumican to the extracellular matrix could recover barrier function in both the fully developed and disturbed regions of vessels treated with siRNA. Figure 5A provides an axial cross-section of the vessel showing the segregation of endothelial cells, smooth muscle, and astrocytes in the model. Radital cross-sections indicated substantial lumican localization to the endothelial periphery in the scrambled control, indicating that the proteoglycan remains in close proximity to the cell membrane. Figure 5B i shows lumican is present throughout the endothelial monolayer, but in the knockdown condition shown in Fig. 5B,ii, lumican is only found in the surrounding smooth muscle cells in the annulus surrounding the endothelium. Therefore, a low concentration of lumican (10 μg/mL) was added to the annulus hydrogel during vessel fabrication of the lumican knockdown condition. Mechanical characterization was conducted to verify that addition of this concentration of lumican did not significantly alter the mechanical or microstructural properties of the hydrogel. Supplemental Fig. 4 indicates that adding 10 μg/mL of lumican did not significantly change the storage and loss modulus of the hydrogel or the pore size and fibrillar diameter as measured by turbidity measurements and scanning electron microscopy, respectively. Addition of the exogeneous lumican substantially increased localization of ZO-1 to the cell–cell junctions in the fully developed region, but was unable to improve ZO-1 localization in the disturbed region (Fig. 5C). Likewise, permeability measurements indicated that the addition of exogenous lumican (1.8 ± 1.1E− 6 cm/s) resulted in no significant difference with the scrambled control (2.2 ± 0.5E− 6 cm/s) in the fully developed region (Fig. 5D). In contrast, the disturbed region of the vessel exhibited increased permeability in both the add-in (7.2 ± 3.9E− 6 cm/s) and scrambled control (6.8 ± 0.9E− 6 cm/s) conditions, indicating that exogenous lumican addition was not sufficient to overcome the barrier disrupting effect of disturbed flow.

## Discussion

The transcriptomic analysis described here demonstrates the importance of fluid shear stress on the expression of genes associated with cell–cell and cell–matrix interactions that mediate blood–brain barrier homeostasis. Our findings show that both the mode of fluid shear stress: fully developed (constant shear stress gradient) versus disturbed (spatially varying shear stress), and the dynamics of the flow: steady versus pulsatile, alter the gene expression of endothelial cells in different regions of a single bifurcation model. Principal component analysis finds distinct clustering between cells exposed to flow and those cultured in static conditions, as well as amongst the cells exposed to various modes of flow. Consistent with previous findings that disturbed fluid flow causes barrier breakdown, cells cultured in static conditions exhibit similar expression patterns to those isolated from areas of altered shear stress (both steady and pulsatile), as indicated by similar clustering along principal component one (PC1). Functional enrichment data show that flow regimes alter the expression of tight junction-associated proteins as well as proteins related to matrix remodeling and structure, indicative of changes to both cell–cell and cell–matrix adhesions. Although further work is required to understand the relative contribution of fluid shear stress to endothelial transcription compared to other factors in the microenvironment of cells including interaction with other cell types like pericytes or immune cells and variation in extracellular matrix composition and structure, these findings reveal that cerebral endothelial cells sense and respond to differences in both temporal and spatial differences in the shear stress applied to the vessel wall.

These results have specific relevance to endothelial function adjacent to bifurcations, where blood flow exhibits complex fluid dynamics and substantial spatial gradients of shear stress. These regions are also where aneurysms preferentially occur, though there are likely a host of factors that dictate whether an aneurysm will form unrelated to fluid dynamics. Nonetheless, the finding that disturbed flow alters gene expression associated with cell–matrix interaction suggests that the shear stress environment at bifurcations may increase the likelihood of aneurysm formation. There is substantial heterogeneity in vascular topology that may make certain people more prone to developing aneurysms: a previous study has shown that wider angles of bifurcations within the middle cerebral artery, which would increase the amount of disturbed flow for a given cerebral blood flow rate, are associated with an increased incidence of aneurysm formation [34]. One limitation of this study is that the applied flow rates are selected to induce disturbed flow within the specific geometry of the 3D blood–brain barrier model, and not to mimic physiological flow. Therefore, further studies can take advantage of recent advances in bioprinting to mimic specific vascular topologies [35] to determine whether similar genes are differentially regulated in cells adjacent to the bifurcation and whether the shear gradient applied to cells in vitro cause barrier breakdown of in vivo vasculature. Still, in steady flow, five of the eight most downregulated genes in cells exposed to disturbed compared to fully developed flow are associated with cell–matrix interactions: either isoforms of matrix metalloproteinases or lumican, a small leucine-rich proteoglycan. Interestingly, MMP-1, MMP-3, MMP-10, and MMP-12 are all downregulated in disturbed flow, which is unexpected due to previous results that MMP-1 and MMP-3 are associated with barrier breakdown [36]. Future work is required to determine whether decreased expression of these enzymes correlate to decreased activity. Moreover, longer time points than 24-h may reveal more information about the effect of flow on cell–matrix interaction and extracellular matrix remodeling.

Less is known about the effect of lumican, the most downregulated gene in the disturbed region in steady flow, on the integrity of the blood–brain barrier. Vessels composed of endothelial cells with lumican expression knocked down by siRNA exhibit increased permeability to 4-kDa dextran following exposure to fully developed flow compared to cells treated with scrambled siRNA controls. Additionally, applying a shear stress gradient representative of disturbed flow decreases the protein expression of lumican in the cells. To our knowledge, these results are the first to show an association between reduced lumican expression and barrier dysfunction in human endothelial cells. Nonetheless, a study found that mice with lumican deficiency exhibited both earlier onset and increased severity of experimental autoimmune encephalomyelitis [37], a disorder characterized by neuroinflammation. Moreover, pericytes, which are known to improve barrier function in endothelial cells, are characterized by increased expression of lumican [38]. More is known about lumican in other tissue systems due to its importance for maintaining the integrity of connective tissue as a mediator of collagen fibrillogenesis, though the evidence related to its effect on the vasculature is unclear. Lumican −/− mice exhibit increased risk of aortic dissection and patients with aortic dissection have increased levels of lumican in their serum [39], though others have found no effect of lumican knockout on blood vessel structure or integrity in mice [40]. Therefore, the results presented here warrant future studies interrogating the effects of lumican downregulation on blood–brain barrier integrity.

Further studies are also required to determine the mechanisms by which lumican expression alters the barrier function of endothelial cells. The results reveal that addition of purified lumican into the surrounding extracellular matrix recovers the barrier function of lumican deficient endothelial cells, but only in the fully developed region of the vessels, suggesting that other mechanisms are involved in barrier disruption in areas of disturbed flow. Moreover, lumican knockdown results in no change in RhoA activation, which our previous studies have demonstrated to be a crucial mediator of shear induced barrier formation in 3D vascular models [41]. The cross-sectional staining indicates substantial lumican staining in the cell periphery, suggesting that the proteoglycan potentially interacts with cell surface receptors. Moreover, a previous study has provided evidence that lumican is incorporated into the glycocalyx of cells [42]. Therefore, future studies may interrogate which receptors interact with lumican to facilitate its effects on the barrier, or determine whether the effects of disturbed flow on lumican downregulation and barrier breakdown are unrelated phenomena.

## Supplementary Information

**Additional file 1: Figure S1.** **A** Photograph of the rheometer setup facilitating application of shear stress to cells on the surface of a collagen hydrogel. HCMEC/D3 monolayers were exposed to fluid shear stress using a 40 mm 1° cone plate to mimic fully developed fluid shear stress on a Peltier plate set to 37 C and 40 mm flat plate to mimic the shear gradient induced by disturbed flow. (B) Predicted absolute shear stress magnitude along the wall of the bifurcation vessel, which was used to determine the shear gradient applied by the flat rheometer plate. **Figure S2.** Full image of the western blots used to quantify lumican expression. (A) Staining with anti-lumican. (B) Stripped and re-probed blot for anti-beta-actin. **Figure S3.** Rho activity assay in cells treated with scrambled siRNA and cells treated with lumican knockdown siRNA. The graph indicates that the mechanistic effects of lumican expression do not alter RhoA activity. n = 3. **Figure S4.** A) Turbidity assay showing the polymerization dynamics for collagen gels containing varying concentrations of lumican: 0.01mg/mL, 0.05 mg/mL and 0.075 mg/mL (n= 3 across three biologically independent experiments). (B–C) Storage and loss moduli of the hydrogels during polymerization (control represents a lumican concentration of zero). (D) Scanning electron microscopy of the hydrogels post-polymerization. E) Quantification of fibril diameter for the hydrogel with the different concentrations of added lumican.

## Data Availability

Raw and processed data presented in this manuscript can be found at the following public data repository: https://dataverse.harvard.edu/dataverse/galielab.
