# Supplementary material for: Transcriptomic analysis of a 3D blood–brain barrier model exposed to disturbed fluid flow
Source: Fluids Barriers CNS. 2022 Nov 24;19:94. doi: 10.1186/s12987-022-00389-x (PMC9700938; doi:10.1186/s12987-022-00389-x)
Supplement: Supplementary file 1 — Additional file 1: Figure S1. A Photograph of the rheometer setup facilitating application of shear stress to cells on the surface of a collagen hydrogel. HCMEC/D3 monolayers were exposed to fluid shear stress using a 40 mm 1° cone plate to mimic fully developed fluid shear stress on a Peltier plate set to 37 C and 40 mm flat plate to mimic the shear gradient induced by disturbed flow. (B) Predicted absolute shear stress magnitude along the wall of the bifurcation vessel, which was used to determine the shear gradient applied by the flat rheometer plate. Figure S2. Full image of the western blots used to quantify lumican expression. (A) Staining with anti-lumican. (B) Stripped and re-probed blot for anti-beta-actin. Figure S3. Rho activity assay in cells treated with scrambled siRNA and cells treated with lumican knockdown siRNA. The graph indicates that the mechanistic effects of lumican expression do not alter RhoA activity. n = 3. Figure S4. A) Turbidity assay showing the polymerization dynamics for collagen gels containing varying concentrations of lumican: 0.01mg/mL, 0.05 mg/mL and 0.075 mg/mL (n= 3 across three biologically independent experiments). (B–C) Storage and loss moduli of the hydrogels during polymerization (control represents a lumican concentration of zero). (D) Scanning electron microscopy of the hydrogels post-polymerization. E) Quantification of fibril diameter for the hydrogel with the different concentrations of added lumican. [file 12987_2022_389_MOESM1_ESM.docx]

Additional file Figures:


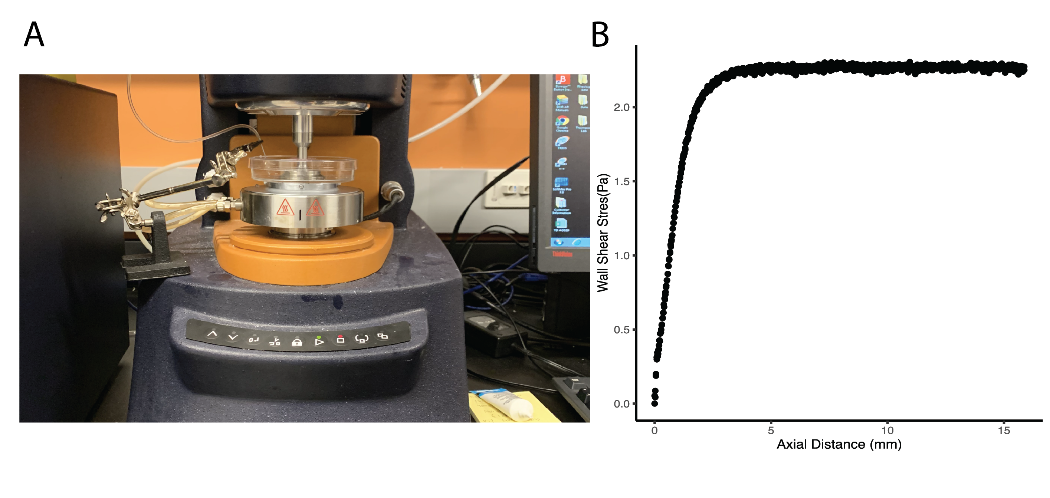


Additional file Figure S1: A) Photograph of the rheometer setup facilitating application of shear stress to cells on the surface of a collagen hydrogel. HCMEC/D3 monolayers were exposed to fluid shear stress using a 40-mm 1- degree cone plate to mimic fully developed fluid shear stress on a Peltier plate set to 37C and 40-mm flat plate to mimic the shear gradient induced by disturbed flow. B) Predicted absolute shear stress magnitude along the wall of the bifurcation vessel, which was used to determine the shear gradient applied by the flat rheometer plate.
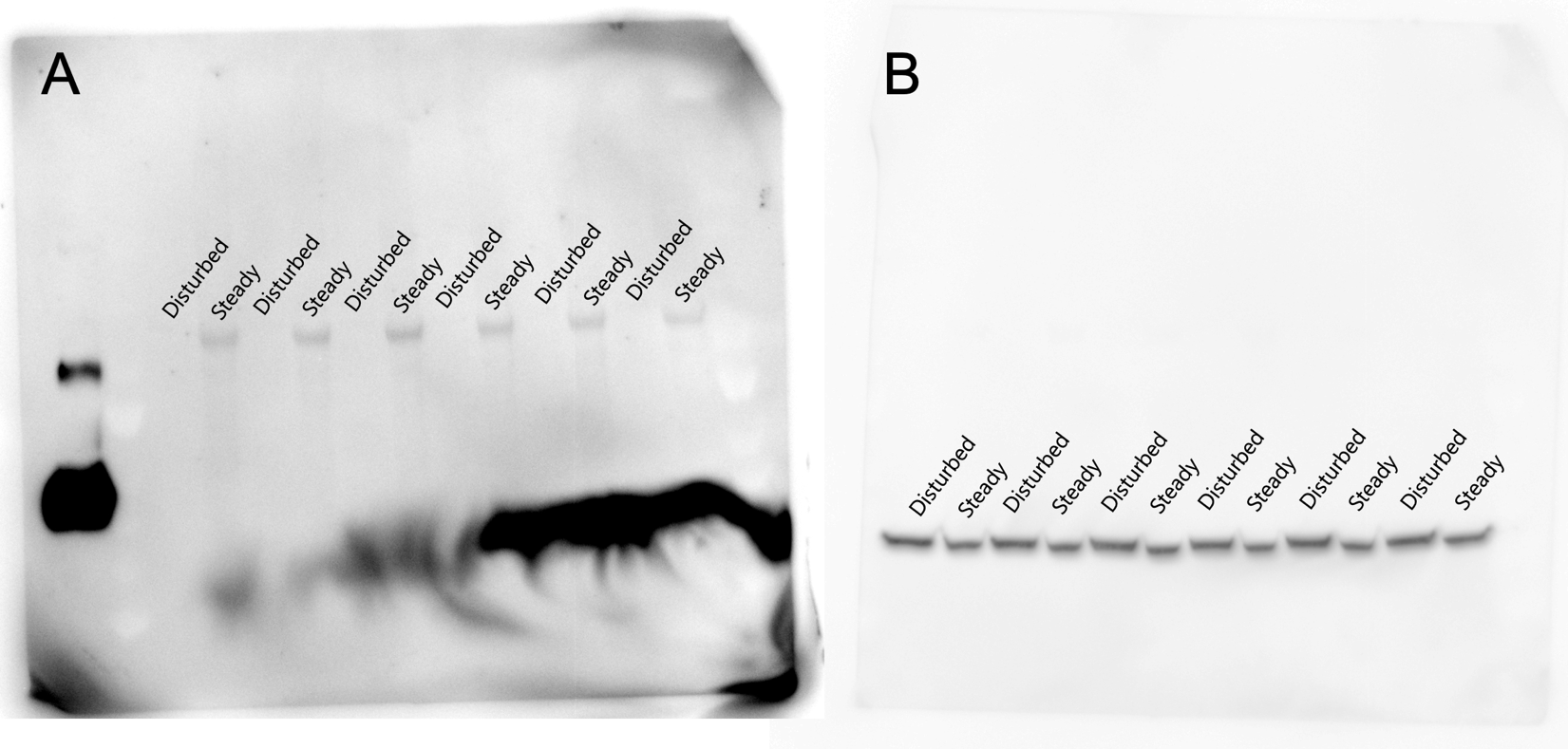


Additional file Figure S2: Full image of the western blots used to quantify lumican expression. A) Staining with anti-lumican. B) Stripped and re-probed blot for anti-beta-actin.


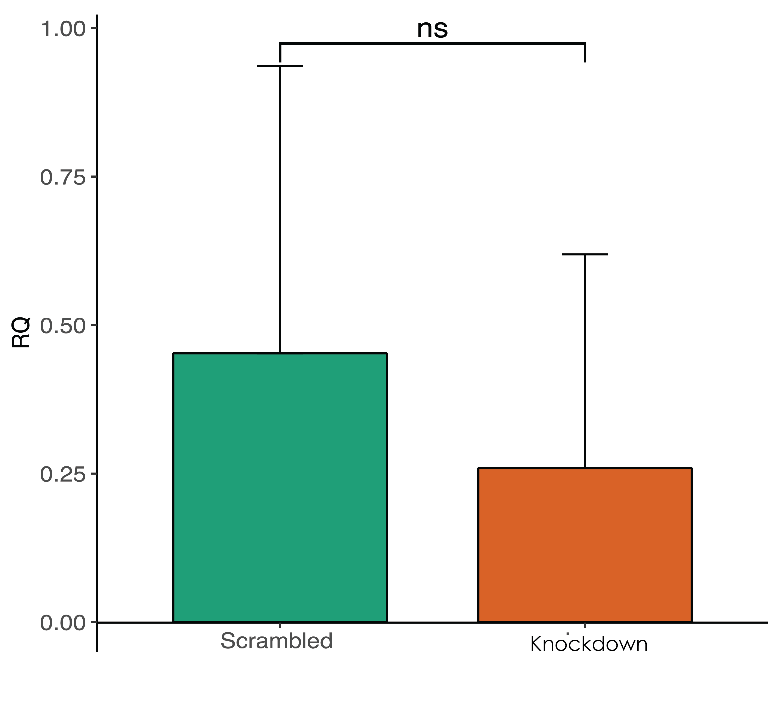


Additional file Figure S3: Rho activity assay in cells treated with scrambled siRNA and cells treated with lumican knockdown siRNA. The graph indicates that the mechanistic effects of lumican expression do not alter RhoA activity. n = 3


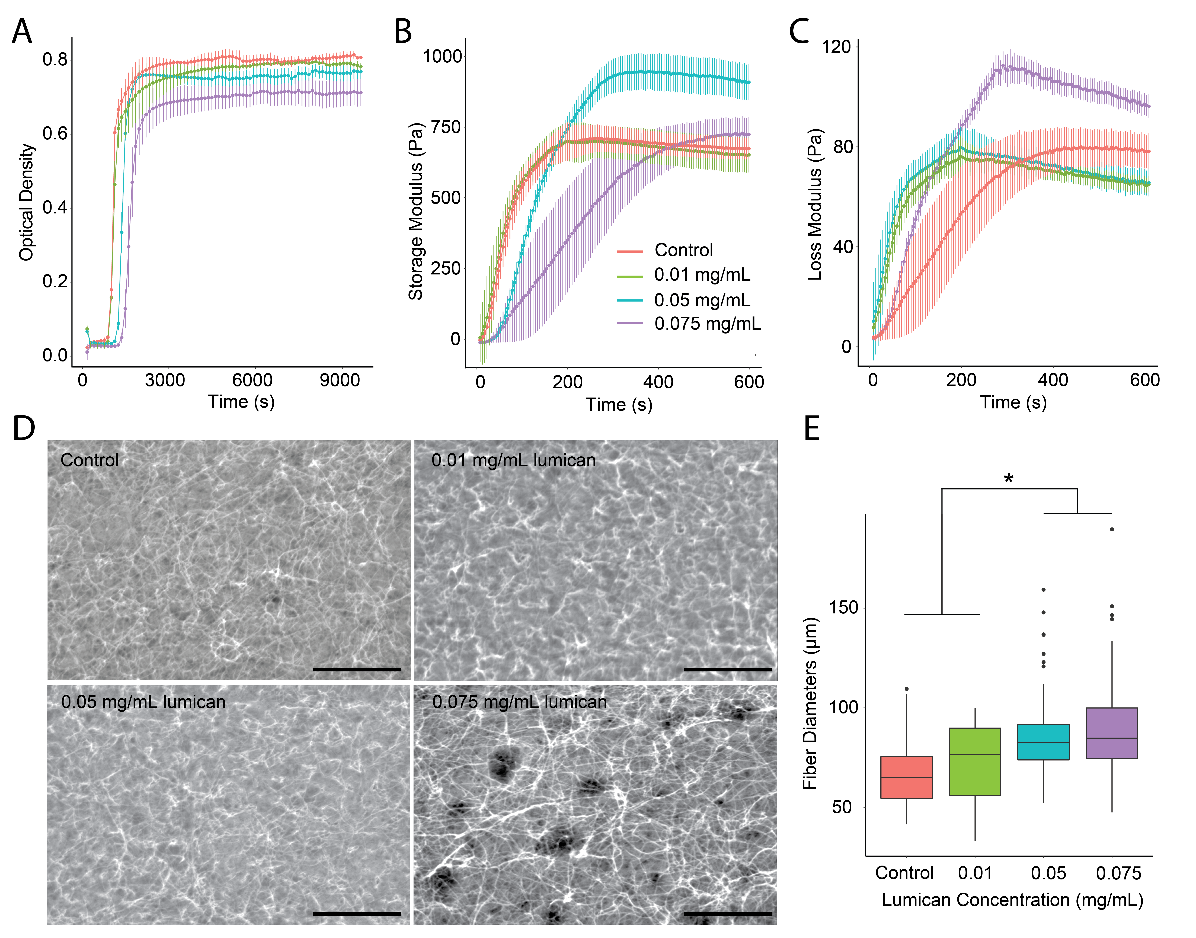


Additional file Figure S4: A) Turbidity assay showing the polymerization dynamics for collagen gels containing varying concentrations of lumican: 0.01mg/mL, 0.05mg/mL and 0.075mg/mL (n= 3 across three biologically independent experiments). B-C) Storage and loss moduli of the hydrogels during polymerization (control represents a lumican concentration of zero). D) Scanning electron microscopy of the hydrogels post-polymerization. E) Quantification of fibril diameter for the hydrogel with the different concentrations of added lumican.
